# Exploring the national burden and challenges in the fight against yellow fever in the Democratic Republic of Congo: a review

**DOI:** 10.1097/MS9.0000000000003730

**Published:** 2025-08-18

**Authors:** Christian Tague, Mayar Moustafa Budair, Maher Ali Rusho, Areeba Aamir Ali Basaria, Rabeea Tariq, Hermann Yokolo, Joshua Ekouo, Farheen Naaz, Dujardin Makeda, Adolphe Karegeya, Mc Juan Muco Mugisha, Calvin R. Wei, Samson Hangi, Elie Kihanduka, Jones Onesime, Excellent Rugendabanga, Aymar Akilimali

**Affiliations:** a Department of Research, Medical Research Circle (MedReC), Goma, DR Congo; b Faculty of Medicine and Dentistry, New Giza University, Giza, Egypt; c Department of Medical Biophysics, University of Toronto, Toronto, Canada; d Department of Medicine, Dow Medical College, Karachi, Pakistan; e Medical College, Deccan College of Medical Sciences, Hyderabad, India; f Faculty of Medicine, University of Rwanda, Kigali, Rwanda; g College of Nursing, University of Utah, Utah, USA; h Clinical Research Department, Rinda Ubuzima Research Organization, Kigali, Rwanda; i Department of Research and Development, Shing Huei Group, Taipei, Taiwan; j International Veterinary Vaccinology Network, The Roslin Institute University of Edinburgh, Edinburgh, United Kingdom; k Global Schistosomiasis Alliance (GSA), London, United Kingdom

**Keywords:** Democratic Republic of Congo, travel, vaccination, yellow fever, yellow fever virus

## Abstract

The Democratic Republic of Congo (DR Congo) is facing a public health emergency due to numerous infectious diseases, predominately yellow fever. Since 2015, numerous outbreaks of the illness have occurred in the country which resulted in detrimental impacts on the population. As of February 2024, the DR Congo has reported over 1,200 suspected yellow fever cases with an 11% case fatality rate. This represents a 22% increase compared to 2021 when 203 confirmed cases were reported with a 9% fatality rate. Although there is no specific medication to treat yellow fever, vaccination is proven to be the most effective method of prevention. Despite national and international efforts to combat the disease through vaccination campaigns, yellow fever continues to pose a significant threat. This is because vaccination efforts are limited by the inadequate infrastructure, poverty, poor sanitation and the presence of rebel groups in the DR Congo. Early diagnosis, the use of mosquito nets and insecticides, as well as raising awareness can furthermore aid in limiting transmission. This review explores the prevalence, diagnosis and prevention methods of yellow fever in the DR Congo, as well as the numerous obstacles faced by the country to eliminate it.

## Introduction

Yellow fever remains a significant public health threat in many developing countries, particularly in the Democratic Republic of Congo (DR Congo). Caused by the yellow fever virus, a member of the Flaviviridae family, the disease is primarily transmitted to humans through the bites of infected Aedes and Haemagogus mosquitoes^[1]^. Although rare, other arthropods such as ticks may also contribute to its transmission. Clinically, yellow fever presents a wide spectrum of manifestations, ranging from self-limiting febrile illness to severe hemorrhagic forms with hepatic dysfunction and multi-organ failure ^[1]^.

**HIGHLIGHTS**

- Yellow fever is a major public health concern in low-middle income countries such as the Democratic Republic of Congo.
- DR Congo reported more than 1,200 suspected cases of yellow fever and it poses a constant threat to public health.
- Yellow fever is a viral tropical disease which is transmitted between humans through mosquito bites, mainly *Aedes aegypti –* a vector from the *Flaviviridae family*.
- The treatment of yellow fever is mainly symptomatic nowadays since there is no specific antiviral medication for it.

**Figure 1.**
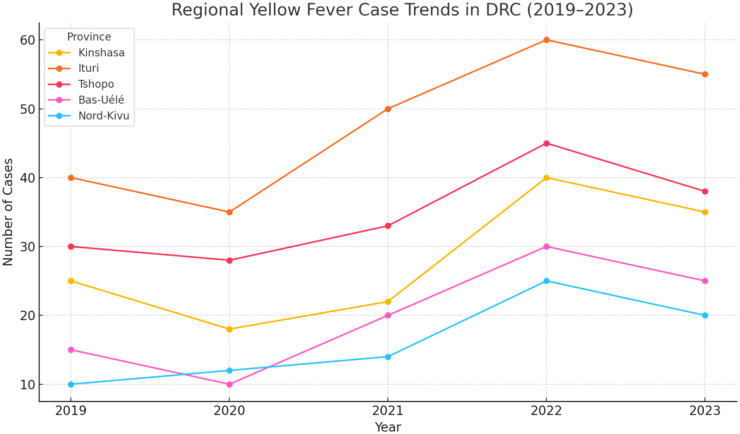
Regional yellow fever case trends in the Democratic Republic of the Congo (2019–2023).

In 2023, the DR Congo reported over 1200 suspected cases, with a concentration of outbreaks in the provinces of Équateur and Tshuapa, as illustrated in Fig. 1 ^[2]^. Despite ongoing efforts by national health authorities and international partners, the country continues to struggle with the effective prevention and control of yellow fever. Several structural and systemic challenges contribute to this persistent threat.

The vast geographical landscape of the DR Congo, coupled with limited healthcare infrastructure, complicates vaccine delivery and outbreak response. During the most recent mass vaccination campaign in 2022, only 60% of the target population was reached, largely due to logistical difficulties, including inadequate transportation to remote areas^[3,4]^. Socio-economic barriers further exacerbate the problem: approximately 74.6% of the population lives on less than $2.15 per day, restricting access to healthcare services and preventive measures^[1]^. Additionally, ongoing armed conflicts in certain regions hinder the implementation of public health interventions and facilitate the spread of infectious diseases, including yellow fever^[5]^.

## Methodology

“This review specifically aims to address the following questions:^[1]^ What is the current burden and trend of yellow fever in the DR Congo?^[6]^ What are the main structural, social, and logistical challenges faced in controlling yellow fever?^[2]^ What evidence-based strategies can be implemented to mitigate its spread?”

This narrative review was conducted using data obtained from PubMed, WHO publications, and reports from the Ministry of Health DR Congo from 2015 to 2024. Search terms included “yellow fever,” “DR Congo,” “vaccination challenges,” “vector control,” and “epidemiology.” Articles were selected based on relevance to yellow fever outbreaks, burden, or management strategies in the DRC. We excluded studies focusing on other arboviruses or countries outside Africa unless contextually relevant.

### Inclusion criteria

- Peer-reviewed articles, official reports, or grey literature published between 2015 and 2025.
- Publications focusing on yellow fever epidemiology, outbreak reports, vaccination efforts, vector control, diagnostics, or health system response specifically in the DRC.
- Articles written in English or French.
- Relevant studies on yellow fever in other African countries if they offered contextual insights or comparative value.

### Exclusion criteria

- Articles focused primarily on other arboviruses (e.g., Zika, dengue) unless they included comparative analysis with yellow fever.
- Publications concerning countries outside Africa, unless directly relevant to yellow fever control strategies applicable to the DRC context.
- Opinion pieces or editorials without empirical or programmatic data.

### Limitations

This narrative review is subject to several limitations that must be acknowledged. First, the study relied primarily on publicly available data and published literature, which may be subject to reporting bias or incomplete surveillance in certain regions of the DR Congo. The lack of standardized case definitions and variations in diagnostic capacity across provinces may also have influenced the accuracy of reported case numbers. Second, while efforts were made to include the most recent and relevant sources, some grey literature and local health data may not have been accessible or indexed in major databases. Third, due to the narrative nature of this review, no formal meta-analysis was conducted, which limits the ability to quantify the burden or the effect size of specific interventions. Lastly, the fast-evolving epidemiological context and ongoing political and humanitarian challenges in the country may affect the timeliness and generalizability of some findings.

## Main text

### Prevalence of yellow fever in the DR Congo

In the DR of Congo, yellow fever poses a constant threat to public health in the tropical subregion. In April 2016, a yellow fever outbreak was declared in the country. Suspected cases and deaths were initially reported in the Central Congo Province bordering Angola, where they first appeared in December 2015. In June 2016, the DR Congo’s Ministry of Health requested assistance from the Centers for Disease Control and Prevention (CDC) to control the outbreak and two mass vaccination campaigns were conducted^[5]^.

**Figure 2.**
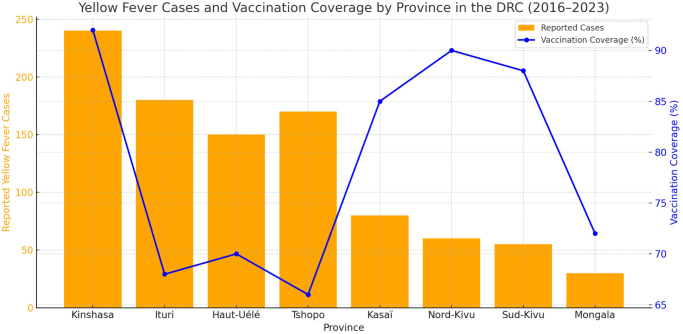
Reported yellow fever cases and vaccination coverage (%) across selected provinces in the Democratic Republic of the Congo, 2016–2023.

During the 2016 outbreak, 410 suspected cases and 42 deaths were reported by August, corresponding to a case fatality rate (CFR) of approximately 10.2%. Subsequent vaccination efforts under the WHO’s EYE Strategy have targeted approximately 62 million people across Africa, including 8 million in DR Congo. However, vaccination coverage in DR Congo remained around 60% during the 2022 campaign, below the 80% threshold necessary to prevent outbreaks. Vaccination campaigns have vaccinated approximately 62 million people in Africa, including a significant proportion in the DR Congo, as part of the World Health Organization (WHO)-led Yellow Fever Epidemic Elimination (EYE) Strategy. However, vaccination coverage remains inadequate, with only 60% of the target population vaccinated during the last mass vaccination campaign in 2022 also shown in (Fig. 2) ^[4]^. This insufficient vaccination coverage is due to several factors, including logistical difficulties in reaching remote areas and limited health infrastructure^[4,7]^.

Regarding the age distribution and sex ratio, in the DR Congo, surveillance data from 2018 to 2023 indicate that approximately 70% of yellow fever cases in DR Congo occur in individuals aged 15–35 years, with males accounting for 55% of cases. This male predominance may relate to occupational exposure and outdoor activities^[4,8]^. In Africa, approximately 77% of cases are observed in people aged 30 years and under. The male to female ratio for suspected cases is often close to 1:1, with a slight male predominance^[9,10]^.

From January 2023 through February 2024, the Ministry of Health DR Congo reported 1,235 suspected yellow fever cases with 136 deaths, yielding an 11% CFR. In comparison, 2021 data showed 203 confirmed and 252 suspected cases with 41 deaths, corresponding to a 9% CFR. The rising CFR over recent years emphasizes the need for enhanced surveillance and treatment capacity^[11]^. These figures show a variation in case fatality rates over the years and highlight the importance of continued surveillance and vaccination efforts to control the spread of yellow fever^[12,13]^. Currently, yellow fever is endemic to 34 countries in Africa and 13 countries in Central and South America.

### Etiology, symptomatology and consequences/complications of yellow fever

Yellow fever is a viral tropical disease which is transmitted between humans through mosquito bites, mainly *Aedes aegypti –* a vector from the *Flaviviridae family*. Animals such as monkeys and gorillas act as reservoirs of the virus. After a period varying between 3–6 days, the virus multiplies in the body and invades the tissues. The first visible symptoms include fever, mild chills, moderate headaches, nausea, vomiting, muscle pain and abdominal pain^[14]^. In the absence of treatment or treatment delay, these manifestations can increase in severity leading to multi-organ failure in the body. This results in liver failure, jaundice, bleeding gums, epistaxis, kidney failure, encephalopathies, cardiac arrhythmias and secondary infections^[14,15]^. Due to the numerous challenges faced by the country, such as poverty and overcrowding, yellow fever has resulted in a devastating impact on the health, economy and socio-cultural aspect of the population^[14,16]^. The poor health infrastructure, lack of hygiene and limited materials prevent the disease from being diagnosed promptly. Consequently, this disease has a very high mortality rate in the DR Congo^[15,17]^.

The decline in the population furthermore impacts the country’s economy, particularly the agricultural and livestock sector due to mass decrease in workforce. Health crises in DR Congo reduce GDP by 2–4% annually^[18]^, with disease outbreaks contributing to the $2.4 billion humanitarian funding gap^[19]^. Regarding the socio-cultural aspect, the high mortality rate generates fear and community disruption with documented psychological impacts. Infectious disease outbreaks cause significant mental health consequences including anxiety, depression, and post-traumatic stress^[20]^.

### Prevention and management of yellow fever in the health context of the DR Congo

Regarding the prevention of yellow fever in the DR Congo, the most effective method is the vaccination of those who reside in health risk areas as well as travelers who may contract it. This measure would greatly limit disease transmission nationally and internationally^[21]^. Global evidence demonstrates yellow fever vaccine efficacy of 95–100% within 10 days of vaccination^[22]^. Between 1970 and 2016, WHO-coordinated vaccination campaigns across Africa are estimated to have averted 51 000–380 000 deaths due to yellow fever. However, vaccination coverage in DR Congo has consistently lagged behind other countries; for example, Brazil achieved 95% coverage in targeted regions by 2015, leading to a 99% reduction in cases, whereas DR Congo’s 2022 coverage was 60%, well below the WHO-recommended 80% threshold^[23]^. Successful programs in Brazil achieved 95% coverage and reduced cases by 99%^[24]^, while DR Congo’s 60% coverage remains below WHO’s recommended 80% threshold for epidemic prevention. Since the disease is transmitted through mosquitos, prevention could be achieved through the distribution and use of insecticide-treated mosquito nets^[25]^. Studies show insecticide-treated nets reduce Aedes aegypti density by 30–50% in urban settings^[26]^, while environmental management can decrease larval indices by 60–80% when consistently applied^[27]^. Measures aimed at vector control could also be implemented, such as the use of insecticides as well as the elimination of breeding sites around homes and the use of repellents when outdoors^[28]^. Another method is avoiding the accumulation of waste and stagnant water which act as a hospitable environment for these mosquitoes^[29]^. Raising awareness amongst the population regarding the importance of vaccination against yellow fever and the danger that its pathology would lead to is imperative^[21,28]^. All these methods will undoubtedly fight against yellow fever effectively and aid in its eradication. Good prognosis of yellow fever begins with early diagnosis. Clinically, there may be acute fever, muscle pain, headaches, chills and jaundice. The diagnosis may be confirmed through PCR (polymerase chain reaction) test which will detect viral RNA^[15]^ serology. The antigen detection test can also be used to identify specific antibodies against the yellow fever virus and highlight the presence of viral proteins in the individual^[7]^. The treatment of yellow fever is mainly symptomatic nowadays since there is no specific antiviral medication for it. It is treated as follows: the fever is relieved with antipyretics such as paracetamol; analgesics to relieve muscle pain and headaches; parenteral or oral rehydration as well as rest and monitoring for possible complications (23,). Cost-effectiveness analysis shows preventive vaccination costs $0.50–2.00 per person versus $200–500 per case treatment, with every $1 invested in prevention saving $8–12 in outbreak response costs^[30]^.

### Obstacles facing the prevention and management of yellow fever in the DR Congo

The Democratic Republic of Congo is a developing country affected by armed conflicts for several decades, which result in numerous challenges relating to the prevention and management of yellow fever. Healthcare workers are unable to supply vaccines and insecticide-treated mosquito nets throughout the country due to numerous reasons^[31]^. The poor road infrastructure and inadequate means of transport limit access to remote regions of the country. The presence of many rebel groups in certain regions such as those bordering Angola and Rwanda make transportation increasingly difficult^[32,33]^. The absence and instability of certain amenities such as electricity and refrigeration appliances pose a challenge in the preservation of vaccines^[29]^. The corruption of surveillance agents at checkpoints furthermore complicating prevention. This is because the passage of unvaccinated individuals cannot be controlled. Another important causative factor is the lack of awareness amongst the population. They lack knowledge regarding the dangers of the disease, the importance of vaccination and the need for continuous sanitation to limit transmission. DR Congo’s vaccination efforts are severely constrained by global vaccine inequity. The global yellow fever vaccine stockpile of only 6 million doses annually cannot meet outbreak demands of 20-30 million doses^[34]^. DR Congo experienced critical delays during the 2016 outbreak when emergency stockpiles were depleted, forcing use of fractional dosing (1/5th dose) to extend supplies^[35]^. Poor patient care is also caused by inadequate technical support in healthcare facilities across the country accompanied by the common practice of self-medication^[17,36]^. Yellow fever dynamics vary significantly across DR Congo’s provinces. Entomological surveys conducted in 2022 showed Aedes aegypti House Indices of 15.2% and Container Indices of 18.4% in Kinshasa, compared with House Indices below 7% and Container Indices below 10% in rural provinces. These elevated indices in urban areas correlate with increased transmission risk^[37]^. Vaccination coverage varies markedly by region: stable western provinces such as Kinshasa report coverage rates of 70–80%, whereas conflict-affected eastern provinces have coverage as low as 30–40% due to insecurity limiting healthcare access. This disparity exacerbates risk in these vulnerable regions^[38]^.

### Recommendations and future perspectives for the eradication of yellow fever in the DR Congo

In order to definitively eradicate yellow fever in the DRC, it is imperative to facilitate the accessibility of vaccination against the disease. It is also vital to raise awareness among the population about the importance of vaccination to avoid the consequences of the illness. The country must set up incentive measures to encourage the population to get vaccinated and equip themselves with adequate health infrastructure. Quality personnel must train 1000 new healthcare workers in vector control by 2026. Additionally, programs must be set up to facilitate the free distribution of mosquito nets to the entire population and develop an initiative to control mosquitos^[13,39,40]^. Establishing a database for monitoring the evolution of the disease through set organizations is also crucial^[40]^. School children should also be educated about infectious diseases, in particular viral diseases like that of yellow fever^[40,41]^. Establish solar-powered vaccine storage in 10 conflict-prone provinces and deploy mobile units with armed escorts for safe access in unstable zones. Also, Global supply of yellow fever vaccine remains limited due to production constraints and stockpile shortages, which can delay outbreak responses. To mitigate these challenges, dose fractioning during emergencies has been successfully implemented to extend vaccine supplies, while improvements in cold chain logistic such as solar-powered refrigerator are critical for maintaining vaccine potency and expanding coverage in remote and resource-limited areas.

## Conclusion

Ultimately, eradicating yellow fever in the DR Congo remains a major challenge exacerbated by geographic, socio-economic and security factors. Despite the efforts made to combat this epidemic, multiple obstacles remain. Nevertheless, solutions exist such as strengthening vaccination campaigns, improving health infrastructure, and distributing insecticide-treated mosquito nets. The establishment of a national surveillance and education program regarding infectious diseases is essential. A multisectoral approach combining national and international efforts can effectively address this major public health problem and hope to eradicate yellow fever in the DR Congo permanently.

## Data Availability

Not applicable.
